# Non-opioid psychiatric medications for chronic pain: systematic review and meta-analysis

**DOI:** 10.3389/fpain.2024.1398442

**Published:** 2024-10-10

**Authors:** Shahana Ayub, Anil Krishna Bachu, Lakshit Jain, Shanli Parnia, Siddhi Bhivandkar, Rizwan Ahmed, Jasleen Kaur, Surya Karlapati, Sakshi Prasad, Hansini Kochhar, Oghenetega Esther Ayisire, Saloni Mitra, Bikona Ghosh, Sushma Srinivas, Sahar Ashraf, Bhavani Nagendra Papudesi, Palash Kumar Malo, Shoib Sheikh, Michael Hsu, Domenico De Berardis, Saeed Ahmed

**Affiliations:** ^1^ Department of Psychiatry, The Institute of Living, Hartford, CT, United States; ^2^ Department of Psychiatry, Atrium Behavior Health, Charlotte, NC, United States; ^3^ Department of Psychiatry, University of Connecticut School of Medicine, Farmington, CT, United States; ^4^ Internal Medicine, Cimpar, S.C., Oak Park, IL, United States; ^5^ Department of Psychiatry, Boston Medical Center, Boston, MA, United States; ^6^ Department of Psychiatry, Liaquat College of Medicine and Dentistry, Karachi, Pakistan; ^7^ Addiction Services Division, Connecticut Valley Hospital, Middletown, CT, United States; ^8^ Department of Psychiatry, Oregon State Hospital, Salem, OR, United States; ^9^ Department of Psychiatry, BronxCare Health System, New York, NY, United States; ^10^ Department of Psychiatry, Maimonides Medical Center, Brooklyn, NY, United States; ^11^ Department of Psychiatry, University of Wisconsin Hospital, Madison, WI, United States; ^12^ Department of Medicine, Bogomolets National Medical University, Kyiv, Ukraine; ^13^ Dhaka Medical College, Dhaka, Bangladesh; ^14^ Permian Basin Campus, Texas Tech University, Lubbock, TX, United States; ^15^ Department of Internal Medicine, Suburban Community Hospital, Philadelphia, PA, United States; ^16^ Centre for Brain Research, Indian Institute of Science, Bengaluru, India; ^17^ Department of Health Services, Srinagar, India; ^18^ Department of Psychiatry, Healing Mind and Wellness Initiative Nawab Bazar, Srinagar, India; ^19^ Department of Psychiatry, Greater Los Angeles VA Medical Center, Los Angeles, CA, United States; ^20^ Department of Mental Health, ASL 4 Teramo, Contrada Casalena, Teramo, Italy; ^21^ Department of Psychiatry, Saint Francis Hospital and Medical Center, Hartford, CT, United States

**Keywords:** chronic pain & fibromyalgia, non-opioid analgesics, pain management (MeSH), SNRI (serotonin-norepinephrine reuptake inhibitors), tricyclic antidepressant drug, gabapentinoids, back pain, psychiatric medication use

## Abstract

**Background:**

The escalating number of deaths related to opioid usage has intensified the pursuit of non-opioid alternatives for managing chronic pain. It's often observed that psychiatric comorbidities coexist in patients suffering from chronic pain. There are a variety of psychotropic medications that have demonstrated effectiveness in treating both psychiatric symptoms and pain. This systematic review and meta-analysis aim to assess the effectiveness of various psychiatric drugs in managing specific types of chronic pain, including fibromyalgia, neuropathic pain, and chronic low back pain.

**Methods:**

A comprehensive search of five major databases was conducted through February 2023 to identify randomized controlled trials (RCTs) that met our inclusion criteria, focusing on outpatients Over 18 years of age with chronic pain. The study assessed the effectiveness of duloxetine, mirogabalin, pregabalin, gabapentin, and tricyclic antidepressants (TCAs), including serotonin-norepinephrine reuptake inhibitors (SNRIs), across various chronic pain conditions such as fibromyalgia, neuropathic pain, and chronic low back pain. The primary outcome measures included pain reduction, improvement in function, and quality of life. Of the 29 RCTs in the systematic review, 20 studies qualified for the meta-analysis. The analysis was stratified by pain type and treatment duration (short-term ≤14 weeks vs. long-term >14 weeks), using Hedge's g standardized mean differences and a random-effects model, along with sensitivity and subgroup analyses.

**Results:**

The overall short-term intervention effect across all studies was significant (SMD −1.45, 95% CI −2.15 to −0.75, *p* < 0.001), with considerable heterogeneity (I^2^ = 99%). For fibromyalgia, both duloxetine and mirogabalin demonstrated substantial efficacy with SMDs of −2.42 (95% CI −3.67 to −1.18, *p* < 0.0001) and −2.10 (95% CI −3.28 to −0.92, *p* = 0.0005), respectively. Conversely, treatments for neuropathic pain and chronic low back pain, including those with amitriptyline and desipramine, did not show significant benefits. The effectiveness of gabapentin could not be conclusively determined due to limited representation in the data. Additionally, no consistent long-term benefits were observed for any of the medications.

**Conclusions:**

While the results of this study underscore the importance of exploring non-opioid alternatives for chronic pain management, particularly in light of the opioid crisis, it is crucial to interpret the findings carefully. Our analysis suggests that certain psychiatric medications, such Duloxetine and mirogabalin demonstrated significant short-term efficacy in fibromyalgia patients. However, their effectiveness in treating neuropathic pain and chronic low back pain was not statistically significant. Additionally, the effectiveness of gabapentin and other medications, such as pregabalin for neuropathic pain, could not be conclusively determined due to limited data and high study heterogeneity. No consistent long-term benefits were observed for any of the drugs studied, raising questions about their sustained efficacy in chronic pain management. These findings highlight the need for further research to understand better the role of psychiatric medications in managing specific chronic pain conditions without prematurely concluding that they are ineffective or unsuitable for these purposes.

## 1 Introduction

While patients find opioids extremely effective for pain control, they carry an associated risk of creating physical dependence, and this has led to the widespread misuse of opioids, and their addiction has developed into a severe public health crisis.

Approximately 21%–29% of individuals administered opioids for chronic pain misuse them, and about 8%–12% develop Opioid Use Disorder (1). Roughly 80% of heroin users began by abusing prescription painkillers (2). Since the year 2000, almost 500,000 people have died as a result of drug overdose (3). Various methods to reduce dependence on opioids, such as restricting their supply, distributing naloxone, and expanding treatment, were found to have only a minor impact on overdose deaths, calling for additional, robust mitigating strategies (4–7). The CDC reported a massive surge in deaths due to opioid overdose between June 2019 and May 2020 (8–10), most likely attributed to increased fentanyl use. Consequently, the USDA has agreed to promote the use of buprenorphine and methadone in patients with opioid use disorder (11, 12). In January 2023, the U.S. government decided to remove the federal requirement for practitioners to possess a DATA Waiver (X-Waiver) to prescribe buprenorphine for the treatment of opioid use disorder (OUD).

Another solution for this opioid epidemic is the utilization of non-opioid medications for pain management. Various studies have found no significant difference in pain management between opioid and non-opioid options (13, 14). There are many non-opioid analgesics on the market now that have better side effect profiles and a lower risk of addiction. Acetaminophen and nonsteroidal anti-inflammatory medicines (NSAIDs) are non-opioid pharmacological choices that have analgesic characteristics for specific illnesses. However, antidepressants and anticonvulsants, the most common adjuvant analgesics (15, 16), not only alleviate pain but also target the underlying psychiatric comorbidities that often accompany chronic pain.

Chronic pain is often highly comorbid with psychiatric disorders (15), such as depression and anxiety, suggesting a shared pathophysiology between these conditions. Psychiatric medications, such as antidepressants and anticonvulsants, work by modulating neurotransmitters like serotonin and norepinephrine. These neurotransmitters play an important role in regulating mood and perceiving pain. Because of this dual effect, these medications could be effective in managing chronic pain, especially in patients who also struggle with psychiatric conditions. For example, studies have shown that duloxetine significantly reduces pain and improves function in patients with fibromyalgia, while pregabalin has been effective in managing pain associated with neuropathy. Psychiatrists often treat patients who suffer from both psychiatric disorders and chronic pain. Addressing the use of psychiatric medications in managing chronic pain is important, as it can help reduce the risk of polypharmacy, improve medication adherence, and minimize the burden of side effects. Involving psychiatry in a multidisciplinary pain management team also allows for better identification of patients who may be developing physical dependence or progressing toward an opioid use disorder. Despite the significant overlap between psychiatric conditions and chronic pain, there are currently no specific guidelines to assist psychiatrists in prescribing these medications for chronic pain management. One considerable advantage of psychiatric medications is their ability to address both the emotional and physical components of pain. Chronic pain often exacerbates conditions like depression and anxiety, which creates a cycle where pain and mental health issues feed into each other. By treating both aspects simultaneously, psychiatric medications can help break this cycle, potentially leading to better overall outcomes for patients. Psychiatric medications tend to have a lower risk profile compared to opioids. These medications are less likely to cause dependence, and their side effect profiles are relatively more favorable than those of long-term opioid use. This makes them a safer alternative, particularly in the context of the ongoing opioid epidemic. Given the serious concerns associated with opioid use, the lower risk profile of psychiatric medications for addiction and overdose makes them a particularly attractive option in the current healthcare landscape. Despite the promising potential of these medications, there remains a significant gap in the guidelines available to psychiatrists for prescribing these drugs specifically for chronic pain management. This study aims to address this gap by systematically reviewing and analyzing the effectiveness of non-opioid psychiatric medications in managing chronic pain conditions such as fibromyalgia, chronic back pain, and neuropathic pain. This study aims to investigate the effectiveness of non-opioid medication in managing certain chronic pain conditions such as fibromyalgia, chronic back pain, and neuropathic pain.

The study focuses on non-opioid psychiatric drugs, including duloxetine, mirogabalin, pregabalin, gabapentin, desipramine, and amitriptyline. By conducting a systematic review and meta-analysis, we seek to update the current knowledge base and contribute to developing safer and more effective pain management strategies.

## 2 Methods

We conducted a systematic review and meta-analysis adhering to the PRISMA Statement guidelines. We searched five databases, including PubMed, Scopus, EMBASE, Web of Science, and Cochrane Library, for articles published within the past decade up until February 2023. We utilized the following keywords: “neuropathic pain,” “fibromyalgia,” and “back pain.” In addition to the initial search terms, we included “radiculopathy,” “sciatica,” “nerve pain,” “central sensitization,” “nociplastic pain,” “musculoskeletal pain,” “discogenic pain,” and “spinal stenosis.” We elected to include Randomized–Controlled Trials conducted in outpatient settings. We focused only on studies where non-opioid psychiatric medications were used as an intervention to treat pain; these include duloxetine, gabapentin, pregabalin, tricyclic antidepressants (TCAs), and serotonin-norepinephrine reuptake inhibitors (SNRIs). Our analysis excluded non-English publications, systematic reviews, case reports, case studies, commentaries, and grey literature. We only considered studies focused on non-acute chronic pain treatment and excluded studies conducted in inpatient settings and those involving alternative treatments like opioid therapy, cannabis, acupuncture, nerve blocks, anti-inflammatory drugs, or corticosteroids.

To identify and control for potential confounding factors, we focused on variables such as patient demographics (e.g., age, gender), baseline pain severity, duration of pain, psychiatric comorbidities, and prior treatments. Studies were included only if they reported sufficient data on these variables, allowing us to perform subgroup analyses where necessary. We also conducted sensitivity analyses to evaluate the robustness of our findings. We used meta-regression to adjust for these potential confounders, ensuring that the impact of these variables on treatment outcomes was minimized.

**Figure 1:**
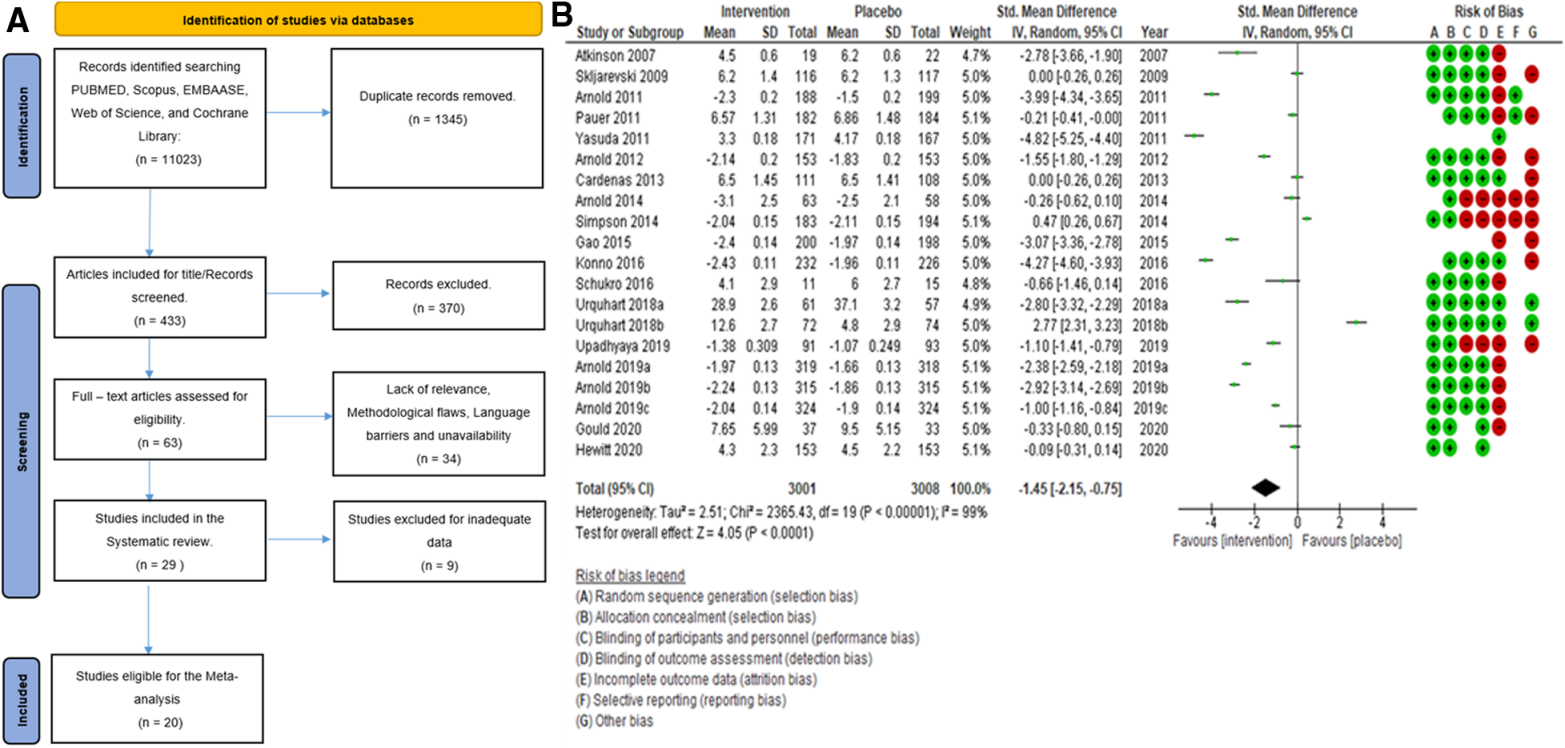
**(A)** Search flowchart. **(B)** Forest plot of the primary analysis of 20 RCT studies with the risk of bias assessment. Green and red circles represent low and high risks of bias.

Search strategy: The initial search, searching through PubMed, Scopus, EMBASE, Web of Science, and Cochrane Library, generated 11,023 citations. After removing duplicates, we had 1,345 studies to screen based on their titles. This process led to the inclusion of 433 citations. We then scrutinized the abstracts, which resulted in the exclusion of 370 citations. The remaining 63 papers were reviewed for eligibility by three authors (SP, SA, and LJ). In case of any disagreements, the 4th and 5th authors (AB and Sehar) stepped in to resolve the disagreements. After applying our stringent inclusion criteria, we selected 29 full-text articles aligned with our research objectives. In addition to our search through five major databases, we employed the snowballing method to enrich our research further. This approach allowed us to discover additional relevant studies and references related to our topic by examining the references in key papers (such as published systematic reviews), including (17–26) Ferraro et al., 2021, Chou et al., 2017, Qaseem et al., 2017, McDonagh et al., 2020, Walitt et al., 2015, Wang et al., 2022, Finnerup et al., 2015, Ferreira et al., 2021, Caruso et al., 2019, and Tort et al., 2012. The PRISMA (27) flow diagram is given below in Figure 1A.

## 3 Meta-analysis

### 3.1 Statistical methods

A total of 29 RCTs were included in the systematic review (Table 1A–C), but only 20 studies (28, 30–34, 36, 38, 43, 44, 48, 50–53, 55, 56, 58) (Atkinson et al., 2007; Skljarevski et al., 2009; Arnold et al., 2011; Pauer et al., 2011; Yasuda et al., 2011; Arnold et al., 2012; Cardenas et al., 2013; Arnold et al., 2014; Simpson et al., 2014; Gao et al., 2015; Konno et al., 2016; Schukro et al., 2016; Urquhart et al., 2018a; Urquhart et al., 2018b; Upadhyaya et al., 2019; Arnold et al., 2019a; Arnold et al., 2019b; Arnold et al., 2019c; Gould et al., 2020; and Hewitt et al., 2020) qualified for the meta-analysis due to the availability of the required data. Arnold et al. (2019) (31) reported the results of three studies separately, and thus, these were considered as three independent studies (Arnold et al., 2019a; Arnold et al., 2019b; and Arnold et al., 2019c) during the analysis. Studies were assessed using the PICO (Population, Intervention, Comparison, and Outcomes) format recommended by the Cochrane Collaboration. Hedge's g standardized mean differences (SMDs) and 95% confidence intervals (C.I.s) were calculated as the continuous study outcome, wherein a negative SMD indicated an effective intervention compared with a placebo. We adopted a random-effects model and used inverse-variance-based DerSimonian and Laird's (DL) estimation method for estimating the summary measure and the heterogeneity variance. In addition, sensitivity analyses were performed by excluding (a) studies with active placebo, such as benztropine, (b) studies with sample sizes < 50 in each study arm, and (c) studies with a high risk of bias. Furthermore, subgroup analyses were performed, and the summary measures were compared for (a) multicenter vs. single-center study settings, (b) neuropathic pain, fibromyalgia vs. chronic low back pain, and (c) duration of the study ≤ 14 weeks vs. study duration > 14 weeks. The impact of heterogeneity was measured using the I2-statistic. The funnel plot was used to check for publication bias. Egger's test was used to check small-study effects. The quality of the studies was evaluated using the Cochrane Risk of Bias assessment tool (59). Analyses were undertaken by intention-to-treat. The statistical significance was checked at a 5% level of significance. Analyses were conducted using the Cochrane RevMan 5.3 and Stata version 17 software.

**Table 1:** **(A)** Studies evaluating fibromyalgia. **(B)** Studies evaluating neuropathic pain. **(C)** Studies evaluating chronic lower back pain.

(A)							
Number	Author	Study design	No. of Patients	Study objectives	Outcome measures	Intervention	Results
1	Pauer et al., 2011 (28)	RCT	736	• Is pregabalin safe to give to patients with fibromyalgia? • Evaluate its efficacy in treating pain and improving functioning	Primary measure: • Endpoint mean pain score from daily pain diaries • Patient Global Impression of Change (PGIC) • Safety assessed via examination for Adverse Events (A.E.). Secondary measures: • Sleep disturbance and sleep quality • Improvement in functioning via Fibromyalgia Impact Questionnaire (FIQ) score • Anxiety and depression via Hospital Anxiety and Depression Scale (HADS).	Pregabalin	• The occurrence of A.E. and the number of patients dropping out of the study increased with an increase in the dose of pregabalin • 450 mg/day pregabalin had modest efficacy in pain, global assessment, and functioning in fibromyalgia • All doses led to statistically significant improvement in sleep
2	Ohta et al., 2012 (29)	RCT	498	Primary: • To assess the effect of pregabalin on fibromyalgia pain Secondary: • To evaluate the safety and tolerability of Pregabalin 300 mg and 450 mg/day • To observe its effects on physical functioning, sleep, and patient's perception of change in quality of life.	Primary measure: • Final assessment mean pain score from daily pain diaries Secondary measures: • Pain scores from Visual Analog Scale (VAS) • PGIC • Improvement in functioning via FIQ score • HADS • Sleep quality and measurement of whether sleep was “Optimal”; • Safety and tolerability assessed via examination for A.E. at each visit.	Pregabalin	• Pain diary scores and VAS scores improved within one week of starting treatment and continued for 15 weeks. • Significant improvement was observed in PGIC and FIQ scores • Improvement was seen in subjective measures of sleep • Both 300 mg/day and 450 mg/day doses of pregabalin were safe, with no serious A.E.s noted • The most common A.E.s were somnolence and dizziness
3	Arnold et al., 2014 (30)	RCT	441	Primary: • Can pregabalin controlled release (C.R.) sustain the initial pain relief felt by patients with fibromyalgia Secondary: • To observe safety, tolerability, and treatment satisfaction with pregabalin C.R. • To assess pregabalin C.R.'s effects on pain severity, functioning, sleep, fatigue, and global assessment.	Primary efficacy endpoint: • Time to loss of therapeutic response (LTR) assessed via daily pain diary Secondary efficacy endpoint: • Daily pain and tiredness diary, with each using an 11-point Numerical Rating Scale (NRS) • Daily sleep diary using subjective sleep questionnaire • PGIC, Medical Outcomes Study–Sleep Scale (MOS-SS), Short-Form 36 Health Survey (SF-36), HADS, FIQ, Multidimensional Fatigue Inventory (MFI), Benefits, Satisfaction, Willingness to Continue (BSW) and Work Productivity and Activity Impairment (WPAI). Safety endpoints: • Summary of A.E. • Physical and neurological evaluations. EKG, laboratory tests, and suicide risk assessments using the Columbia–Suicide Severity Rating Scale (C-SSRS).	Pregabalin	• Pregabalin C.R. was more effective than placebo. • In the single-blind phase, patients on pregabalin C.R. exhibited improvements in secondary measures. • In the double-blind phase, the only statistically significant secondary measure was the patient's reporting of treatment benefits. • Higher frequency of A.E., but fewer discontinuations were found in the double-blind phase. • Once-daily dosing may have led to better adherence.
4	Arnold et al., 2019 (31)	RCT	3,864	• Three identical RCTs compared pregabalin (75 mg–150 mg/day), mirogabalin (15 mg/day), and mirogabalin (30 mg/day) to an active placebo in patients with fibromyalgia • The study also evaluated the safety and tolerability of the two different doses of mirogabalin • RCT-completing patients were offered to join a 52-week open-label extension phase to assess the long-term efficacy and safety of mirogabalin.	Primary efficacy endpoints: • Change in average daily worst pain score (ADPS) from baseline to week 13 Secondary efficacy endpoints: • > 30% or >50% reduction in ADPS, PGIC and FIQ Exploratory objectives: • Changes in ADPS in mirogabalin vs. pregabalin group • Pain via BPI-SF; • Fatigue using Multidimensional Fatigue Inventory–20 (MFI-20) • Depression and anxiety through HADS • General health and quality of life using SF-36 and EQ-5D • Sleep using MOS-SS and pain-related sleep interference using Average Daily Sleep Interference Scores (ADSIS). Safety measures • A.E. monitoring • Physical examinations, vitals, laboratory tests, suicide risk using C-SSRS.	Pregabalin and mirogabalin	• Mirogabalin could not exhibit a statistically significant reduction in ADPS score or scores for secondary outcome measures from baseline at both doses. • Pregabalin exhibited inconsistent results, with only a statistically significant reduction in ADPS scores in Studies B and C. • In all three studies, a significant reduction was observed in pain-related sleep interference • Mirogabalin exhibited statistically significant improvements in fatigue at both doses. It also showed a reduction in BPI-SF pain severity scores. • The rates of A.E. were similar for both doses of mirogabalin and were comparable to pregabalin. However, rates of discontinuation were slightly higher in the higher-mirogabalin-dose group. • The rates of A.E. were similar for both doses of mirogabalin
5	Arnold et al., 2012 (32)	RCT	308	• To assess the effectiveness of Duloxetine 30 mg/day in patients with fibromyalgia • To evaluate the safety and tolerability of duloxetine 30 mg/day	• BPI-SF • PGIC • FIQ score • Numerical scores for pain, depression, anxiety, health outcomes, and safety	Duloxetine	• Fibromyalgia patients taking 30 mg/daily of duloxetine did not experience any improvement in their pain after three months of treatment.
6	Arnold et al., 2011 (33)	Secondary analysis of an RCT	530	• Examine the effectiveness of duloxetine in improving fatigue in patients who have fibromyalgia.	• MFI • BPI average pain • Numerical scales to rate anxiety, depressed mood, sleep difficulties, and musculoskeletal stiffness	Duloxetine	• Patients receiving duloxetine 60–120 mg/day had a significant improvement in fatigue compared to the control group within four weeks. • The fatigue improvement was sustained for up to 24 weeks, with improvement in both physical and mental aspects of fatigue. • Duloxetine also decreased anxiety, depressed mood, and sleep difficulty.
7	Upadhyaya et al., 2019 (34)	RCT	184	• Effectiveness and safety of duloxetine 30–60 mg/day treatment in adolescents with juvenile fibromyalgia.	• 24 h change in mean pain severity of BPI • BPI severity and interference scores • Treatment response (≥30%, ≥ 50% reductions on BPI average pain severity) • Pediatric Pain Questionnaire • Clinical Global Impression of Severity: Overall and Mental Illness scales • Functional Disability Inventory: child and parent versions • Children's Depression Inventory • Multidimensional Anxiety Scale for Children • Safety and tolerability	Duloxetine	• There was no statistically significant difference in the mean change of the 24-hour average pain severity of the BPI among the placebo and duloxetine groups. • However, treatment with duloxetine had a significant response compared to placebo for one of their secondary endpoints: 30% and 50% reductions in average pain severity.
8	Chappell et al., 2009 (35)	RCT	350	• To investigate the safety and effectiveness of duloxetine (30, 60, 120 mg/day) in patients experiencing fibromyalgia.	• BPI- Modified Short Form • FIQ • Patient's and Clinical Global Impression of Severity • The mean of the 18 tender points pain thresholds • The number of tender points with a low threshold (<4 kg cm^2^) • The Sheehan Disability Scale Global Functional Impairment Score	Duloxetine	• In the subset of patients who did not have a clinically significant reduction in pain intensity after eight weeks of treatment, no increased efficacy benefit was observed with duloxetine 120 mg daily compared with duloxetine 60 mg daily.

(B)							
Number	Author	Study Design	No. of Patients	Objective of Study	Outcome Measures	Intervention	Results
1	Simpson, D. M., et al. (2014) (36)	RCT	377	• To evaluate the safety and tolerability of flexible-dose pregabalin (450 mg–600 mg/day) in patients with HIV-associated distal sensory polyneuropathy (DSP) • To compare its efficacy with a placebo • 6-month open-label extension study to test the safety and tolerability of long-term treatment	Efficacy outcome measure Primary: • Change in daily average pain score (11-point NRS scale) Secondary: • PGIC and Clinician Global Impression of Change (CGIC); • Neuropathic pain via weekly NRS, Brief Pain Inventory short form (BPI-SF), and Neuropathic Pain Symptom Inventory (NPSI) • Sleep via NRS sleep scale and MOS-SS • Daytime activity via Actiwatch measures actigraphy data • Quality of life via Work Productivity and Activity Impairment Specific Health Problem (WPAI-SSP) questionnaire and SF-36 • Mood via HADS. Safety and tolerability outcome measure: • Monitoring of A.E. • Laboratory measurements, toxicology screenings, pregnancy tests, vitals, neurological examination, general history, and physical examination Open-label Phase • Pain scores on VAS • PGIC • (WPAI-SSP) and SF-36	Pregabalin	• Both RCT and Open-label extension exhibited no statistical difference between pregabalin and placebo in mean pain scores. • No differences were observed in sleep measures between pregabalin and placebo. • Pregabalin was well tolerated, with <2% patient's reporting A.E.s • Incidence of A.E.s like dizziness and drowsiness was lower than other RCTs
2	Kim, J. S., et al., 2011 (37)	RCT	219	• To evaluate the tolerability and safety of pregabalin (150 mg–600 mg/day) in patients with central post-stroke pain (CPSP) • To compare the efficacy of pregabalin versus placebo in pain relief and other quality-of-life measures in patients with CPSP	Primary endpoint: • Mean pain score of the last seven scores of the daily pain rating scale Secondary endpoint: • MOS-SS and Daily Sleep Interference Scale (DSIS) • Neuropathic pain measures like NPSI, Quantitative Assessment of Neuropathic Pain (QANeP), and Short-form McGill Pain Questionnaire (SF-MPQ); • Quality-of-life measures like PGIC, CGIC, Euro Quality of Life (EQ-5D), and HADS.	Pregabalin	• No significant difference was observed in pain scores between pregabalin and placebo. • Pregabalin produced sizeable pain relief in the first eight weeks with no loss in pain afterward, while placebo led to gradual pain reductions over time. • In secondary measures, pregabalin exhibited a statistically significant reduction in anxiety scores and several sleep interference scores. • Statistically significant improvements were seen in CGIC • AE rates were similar to other studies
3	Diana D Cárdenas et al., 2013 (38)	RCT	404	• To assess the efficacy of pregabalin in patients with chronic central neuropathic pain due to spinal cord injury (SCI) • This study also evaluated the safety and tolerability of pregabalin in this population	Efficacy outcome Primary: • Duration-Adjusted Average Change (DAAC) in pain (weighted average of available and missing pain scores) Secondary: • Mean pain score • PGIC score • Pain-related sleep interference score (from an 11-point scale sleep diary) • MOS-SS • HADS Safety and tolerability: • A.E. reporting • Vitals, EKG, and laboratory tests.	Pregabalin	• Pregabalin improved DAAC in pain over placebo and led to statistically significant improvement in mean pain scores, PGIC score, and pain-related sleep interference scores • Improvements in mean pain and pain-related sleep interference with pregabalin were pronounced in week 1 • 67% of patients utilized more than 450 mg/day dose of pregabalin, indicating SCI patients may need higher doses • A.E. were observed more frequently, with 67% experiencing > 1 A.E. • Somnolence was observed more frequently than in other studies.
4	Satoh, J., et al., 2011 (39)	RCT	317	• To identify the efficacy of pregabalin in providing relief in neuropathic pain associated with diabetic peripheral neuropathy (DPNP) in Japanese patients • To evaluate its safety and pharmacokinetics in the Japanese population	Efficacy outcome Primary: • Change from baseline in weekly mean pain score (from daily pain diary using 11-point NRS) Secondary: • Weekly mean pain score • Responder rates • SF-MPQ • Weekly mean sleep interference score (from 11 points NRS) • MOS-SS • SF-36 • Patient impression of subjective symptoms • PGIC and CGIC. Safety and tolerability • A.E. reporting • Physical examinations and laboratory tests. Pharmacokinetics: • Blood samples at weeks 8 and 13	Pregabalin	• Pregabalin (300 mg and 600 mg) led to a statistically significant reduction in mean pain scores at the endpoint from baseline, caused a superior reduction in weekly mean pain scores, had a higher response rate (at both doses), and significantly decreased sleep interference. • Statistically significant improvements were also seen in some subscales of MOS-SS and SF-36. • Pregabalin 300 mg led to improvement in numbness and pain, and 600 mg led to improvements in paresthesias. • PGIC scores favored pregabalin 600 mg, while CGIC scores favored both groups over placebo • A.E.s were common, were similar to rates observed in previous studies, and were seen more commonly in pregabalin 600 mg patients. • The pharmacokinetic profile of pregabalin was within anticipated limits.
5	Julia Boyle et al., 2012 (40)	RCT	83	• To compare amitriptyline (50 mg–75 mg/day), pregabalin (300 mg–600 mg/day), and duloxetine (60 mg–120 mg/day) in their ability to manage chronic DPNP • To further evaluate the effects of these three treatments on sleep (quantity and quality), cognitive functioning, and quality of life in patients with DPNP	Primary outcome: • Subjective pain is assessed using Brief Pain Inventory (BPI) Secondary outcome: • SF-36 • Subjective pain was assessed using BPI and VAS adapted from the SF-MPQ • Sleep evaluated using VASs like the Leeds Sleep Evaluation Questionnaire, Linear Analog Rating Scale, and Karolinska Sleepiness Scale • Polysomnography (PSG) records are manually staged and evaluated using Rechtschaffen and Kales criteria • Cognitive functioning sensory-motor and psychomotor speed (continuous tracking task and choice reaction time task); central nervous system (CNS) arousal and information processing functions (critical flicker fusion), Stroop task, digit symbol substitution test (DSST) and working and explicit memory tasks [immediate and delayed word recall and Sternberg short-term memory scanning task (STM)]. Safety measures: • A.E. monitoring • Physical examinations, vitals, EKG, and laboratory tests.	Pregabalin, amitriptyline, duloxetine	• No statistically significant difference in subjective pain scores and subjective sleep scores was observed, and no one treatment was found superior to others. • Duloxetine reduced sleep efficacy, reduced total sleep duration, increased wake after sleep onset, and caused more noticeable loss of REM sleep. • Pregabalin reduced the periodic limb movement index and increased the apnea and hypopnea index. • Daytime functioning—duloxetine and amitriptyline improved reaction time; duloxetine improved CNS arousal and information processing, and pregabalin hindered daytime functioning by increasing tracking errors. • Pregabalin group had the most AE. No significant changes of note were observed in safety assessments, nocturnal blood glucose—a small but significant increase caused by duloxetine and a decrease caused by pregabalin.
6	Padmini Devi et al., 2012 (41)	Randomized, open-label, comparative study	152	• To establish the safety of gabapentin, duloxetine, and pregabalin in patients with painful diabetic peripheral neuropathy (DPNP) • To compare the efficacy of these medications in treating DPNP	Primary measure: • Pain severity as measured on 11-point VAS in daily diaries Secondary measure: • Monthly mean sleep interference scores from daily sleep diaries measured on an 11-point scale; • PGIC and CGIC. Safety measures: • Adverse Drug Reactions (ADR) reported by patients	Pregabalin, gabapentin, duloxetine	• All three treatments led to a significant reduction in pain from baseline, with pregabalin exhibiting a sharper decline at week 4 • All three groups saw a reduction in sleep interference, with pregabalin again showing more improvement than the other two. • Significant improvements in PGIC and CGIC scores were observed. While there were no differences among groups regarding CGIC, pregabalin and gabapentin caused a higher response in PGIC scores. • ADRs reported were mild.
7	Rauck R., et al., 2013 (42)	RCT	421	• This study aimed to identify ideal gabapentin enacarbil dosages that can be used in future diabetic peripheral neuropathy trials	Primary measure: • Mean 24 h average pain intensity score based on an 11-point Pain Intensity Numerical Rating Scale (PI-NRS) Secondary efficacy measure: • PGIC and CGIC • SF-MPQ and neuropathic Pain Scale (NPS; • Profile of Mood State (POMS) and SF-36. Safety and tolerability assessments: • A.E. monitoring • Physical examinations, vitals, EKG laboratory tests, etc.	Gabapentin enacarbil, pregabalin	• No significant difference between gabapentin enacarbil, pregabalin, and placebo • No improvement in secondary efficacy measures was seen with gabapentin enacarbil • The rates of A.E. were similar in the gabapentin enacarbil and pregabalin groups, with no consistent patterns of A.E. (except weight gain in the gabapentin enacarbil group)
8	Yasuda et al., 2011 (43)	RCT	339	Investigate the efficacy and safety of duloxetine in Japanese patients with diabetic neuropathic pain (DNP)	Primary measure: • Mean 24-hour average pain severity score on the 11-point scale Secondary measures: • Numerical Rating Scale • Patient's Global Impression of Improvement (PGI-I) • Brief Pain Inventory (BPI).	Duloxetine	• The trial demonstrated that duloxetine 40 mg and 60 mg were safe and well tolerated. • The once-daily dosing of duloxetine was superior to placebo in improving DNP in Japanese patients.
9	Y Gao et al., 2015. (44)	RCT	405	Assess the efficacy and safety of 60 mg of duloxetine once daily compared with placebo in Chinese patients suffering from DPNP.	• Michigan Neuropathy Screening Instrument • Brief Pain Inventory–Modified Short Form	Duloxetine	The study trial suggests that duloxetine-treated patients showed significantly greater pain relief than placebo-treated patients over the 12-week study period.
10	Yasuda, H., et al., 2016 (45)	RCT	258	Examine the long-term efficacy and safety of duloxetine in the treatment of Japanese patients with diabetic neuropathic pain.	• Pain using Brief Pain Inventory severity and interference • Quality of life using Patient's Global Impression of Improvement • Safety using adverse events, vital signs, metabolic measures	Duloxetine	Long-term duloxetine therapy for diabetic neuropathic pain is effective and has an acceptable safety profile.

(C)							
Number	Author	Study Design	No. of Patient	Objective of Study	Outcome Measures	Intervention	Results
1	J Kalita, 2014 (46)	Open-label RCT	200	• To compare the efficacy of amitriptyline (AMT) and pregabalin (P.G.) in patients with chronic lower backache (CLBA) • To establish the safety of these medications in a patient with CLBA (backache of at least three months duration with no specific etiology)	Primary outcome: • Pain measured by VAS score (0 to 10) Secondary outcome: • Improvement in disability measured using Oswestry Disability Index (ODI) version 2 • Safety measures using A.E. monitoring, physical examinations, vitals, EKG and laboratory tests, MRI, etc.	Pregabalin, amitriptyline	• VAS and ODI scores improved in both groups, although improvement in the amitriptyline group was superior at week 14 • Adverse events were more common in the pregabalin group (21), with vertigo being the most common A.E. • Patients were divided into three subgroups: localized CLBA, CLBA with radiculopathy, and CLBA with lumbar canal stenosis (LCS). Depending on the subgroup, there were no differences in pain relief and disability score reduction. • No differences were observed between patients with and without neurological deficits regarding pain relief or disability reduction. • The dose of medication did not differ between those who experienced pain relief and those who did not, nor did it affect the degree of disability reduction.
2	J Hampton Atkinson, 2016 (47)	RCT	423	• This research is a randomized, 12-week, parallel-group study aiming to study the effectiveness of gabapentin on chronic low back pain. • To tackle shortcomings in back pain analgesic studies such as sampling, study duration, and confounding by depression and chronic pain	• The primary outcome measure was pain intensity as determined by the Descriptor Differential Scale (DDS) • Secondary outcomes were evaluated for everyday functioning using the ODI, Version 2.0, and mood assessment by the Beck Depression Inventory-II. • Safety measures were taken at study entry, such as A.E. monitoring, physical examinations, and lab tests, with the UKU Side Effect Rating Scale used for further assessment.	Gabapentin	• Pain intensity decreased for both the gabapentin and placebo groups, with no statistically significant difference between the two groups • There was also no statistically significant difference between the two groups in secondary measures • 49 out of 55 patients on gabapentin experienced A.E.s
3	Skljarevski, V et al., 2009 (48)	RCT	404	Examine the efficacy of duloxetine 20 mg, 60 mg, and 120 mg O.D. compared with placebo in patients with CLBP.	• Mean 24-hour average pain • Roland–Morris Disability Questionnaire (RMDQ-24) • Patient's Global Impressions of Improvement (PGI-I) • Brief Pain Inventory (BPI) • Safety and tolerability	Duloxetine	• The study revealed that in the treatment of CLBP, duloxetine at three different doses did not significantly reduce average weekly pain compared to a placebo. • However, duloxetine showed superiority over placebo in multiple secondary measures and was well tolerated.
**4**	Williamson O.D., et al., 2014 (49)	*post hoc* Analysis of RCT	780	To determine how long it takes to treat osteoarthritis (O.A.) knee pain and chronic low back pain (CLBP) with duloxetine before considering a change in medication strategy	• Pain severity is recorded daily in patient diaries using an ordinal 11-point NRS (0 = no pain to 10 = most severe pain) • Kaplan–Meier methods	Duloxetine	• The trial demonstrated less than 10% reduction in pain after four weeks of treatment in patients taking duloxetine for O.A. or CLBP. • The possibility of achieving even moderate pain reduction by the end of 12 weeks is limited.
**5**	Schukro, R.P., et al., 2016 (50)	RCT	41	Evaluate the efficacy of duloxetine in the treatment of CLBP patients with neuropathic leg pain.	• Mean VAS score during the last week of treatment in each phase (VAS week 4)	Duloxetine	• The study revealed that duloxetine was superior to placebo in treating CLBP with neuropathic leg pain.
6	Shinichi Konno, 2016 (51)	RCT	458	Assess the efficacy and safety of duloxetine 60 mg monotherapy in Japanese patients with CLBP	Primary efficacy measure: • BPI average pain score from baseline to week 14 Secondary efficacy measures: • BPI pain (worst pain, most minor pain, pain right now) • Patient's Global Impression of Improvement, Clinical Global Impressions of Severity • RMDQ • Safety and tolerability.	Duloxetine	The trial's findings suggest that 60 mg of duloxetine once daily is effective and well-tolerated in treating Japanese patients with CLBP.
7	Donna M. Urquhart, et al., 2018 (52)	RCT	150	Evaluate the efficacy of low-dose amitriptyline compared to placebo in reducing chronic low back pain	Primary outcome measure: • Pain intensity using a Visual Analogue Scale (VAS) of 100 mm. Secondary outcome measures: • RMDQ • Absenteeism and hindrance in paid and unpaid work performance using the Short Form Health and Labor questionnaire (SFHQ).	Amitriptyline	• The trial suggests that low-dose amitriptyline may be effective in treating CLBP.
8	J. Hampton Atkinson et al., 2007 (53)	RCT	121	Evaluate: • the feasibility of conducting a controlled-concentration study of a norepinephrine (desipramine) and a serotonin reuptake (fluoxetine) inhibitor • the relationship between achieved concentrations and analgesic response in chronic back pain	Primary outcome measure • Pain intensity at present reported on the Descriptor Differential Scale (DDS) Secondary outcome measure: • Impairment in activities of daily living as determined by the Roland and Morris Disability Scale.	Desipramine at 50 mg, 110 mg, and 150 mg; and fluoxetine at 100 mg, 200 mg and 400 mg	• The study revealed a significant overlap of assigned and achieved concentrations related to drug intolerability. • There was a significant reduction in pain intensity and improvement in everyday function at low concentrations of desipramine compared with placebo, higher concentrations of desipramine, and all concentrations of fluoxetine.
9	Jurg Schliessbach et al, 2018 (54)	RCT	50	Evaluate the effect of a single dose of imipramine for chronic lower back pain.		A single dose of imipramine	• The trial results demonstrate that a single oral dose of imipramine did not produce an analgesic effect within 2 h in chronic lower back pain patients. Mood and sleep modulation were the significant factors compared to the direct analgesic effect.
10	Gould HM et al., 2020 (55)	RCT	141	• To compare the independent and combined effects of desipramine (serum concentration 15–65 ng/ml) and CBT (6 sessions over eight weeks) to an active placebo in patients with chronic low back pain using a 12-week-long, 4-arm RCT • To identify the rate of clinically meaningful improvement (>30% improvement) in pain intensity or disability	Efficacy measures: • DDS • RMDQ. Safety measures: • A.E. monitoring • Interview with study physician • UKU Side Effects Rating Scale.	Desipramine, CBT	• Four treatment arms (Desipramine alone, Desipramine + CBT, CBT + active placebo, and active placebo) saw a statistically significant reduction in pain measures. • The desipramine group had the most participants, and it reported a > 30% reduction from baseline DDS scores. • CBT group participants reported the highest number with >30% reduction in RMDQ scores • More AEs were reported in the desipramine group (1 serious A.E. and 70.6% other A.E.), but no differences between desipramine and placebo groups were observed. • Dropout rates for all four treatment arms were similar.
11	Horne AW et al., 2021 (GAPP2 trial) (56, 57)	RCT	306	• To evaluate the efficacy of gabapentin in women with chronic pelvic pain and no apparent pelvic pathology • To further evaluate the safety of gabapentin in these patients	The primary outcomes included worst and average pain scores measured through NRS. The secondary outcomes included examination of patients with a reduction in pain scores of 30% or 50%, general quality of life, fatigue, work impairments, neuropathic features of pain, pain-related cognitions, distress, sexual functioning, adherence to treatment, and safety measures. • Questionnaire • Treatment adherence. Safety measures: • A.E. monitoring	gabapentin	• The study found that gabapentin did not enable a statistically significant improvement in pain scores when compared to a placebo. The adherence rates for gabapentin were similar to those of the placebo, but more adverse effects were observed in the gabapentin group, including dizziness, drowsiness, and visual disturbances.

### 3.2 The review of the literature

Fibromyalgia is a chronic disorder that is primarily characterized by the presence of generalized pain throughout the body. Pain is only one symptom of its complex, multifaceted symptomatology. When treating fibromyalgia, it is essential to adopt a comprehensive approach. A treatment plan may include thorough assessments, goal-setting, education on the condition, pharmacological and non-pharmacological interventions, alternative, complementary therapies, and lifestyle modifications (60, 61). We have investigated several pharmacological non-opioid treatments for fibromyalgia, such as pregabalin, mirogabalin, and duloxetine. Studies have shown that some of these medications are effective in alleviating pain related to fibromyalgia and also improving overall functioning (28, 29). Pauer et al. (28) conducted a 14-week study investigating the use of pregabalin for its efficacy, safety, and functioning in 736 individuals with fibromyalgia. The research showed that 450 mg/day of pregabalin significantly reduced pain scores and improved sleep and function. Ohta et al. investigated the efficacy of pregabalin in Japanese patients. The subjects started at 150 mg/day and subsequently increased to a maintenance dose of either 300 or 450 mg/day over 15 weeks. The results show that 450 mg/day of pregabalin effectively reduced pain and improved sleep and functioning. There were, however, minor adverse effects, including dizziness and sleepiness. The findings of this study support the potential use of pregabalin as a viable and safe treatment option for fibromyalgia patients. In a centers-based study, Arnold and colleagues (2014) (30) conducted a study to evaluate the safety and efficacy of a controlled-release (C.R.) version of pregabalin in patients with fibromyalgia. The study initially included 441 participants who underwent a 6-week single-blind treatment phase with pregabalin C.R., followed by a 13-week double-blind phase where patients were randomized to continue with pregabalin C.R. or switch to a placebo.

The key outcome measured was the time to loss of therapeutic response (LTR), defined as a less than 30% pain reduction from the single-blind phase baseline or discontinuation due to lack of efficacy or adverse events. The results show that the median time to LTR was significantly longer for patients in the pregabalin C.R. group (58 days) compared to the placebo group (22 days). By the end of the trial, 54.0% of patients in the pregabalin C.R. group experienced LTR, which is lower than the 70.7% in the placebo group, indicating that a more significant proportion of patients in the pregabalin C.R. group maintained their therapeutic response. Dizziness and sleepiness were reported but were generally mild to moderate in their rating.

Another study exploring mirogabalin compared its efficacy with pregabalin in a 13-week randomized trial followed by a 52-week open-label extension (31). In this study, 1,293 patients were given a placebo, 1,270 pregabalin (150 mg twice daily), and 1,301 received mirogabalin (15 mg once or twice daily). The results show that pregabalin significantly reduced pain, while mirogabalin at 15 mg once or twice daily did not produce comparable outcomes. Duloxetine has been studied for its effects on fatigue and pain severity, showing improvements in both outcomes (32, 35). Research studies have investigated duloxetine in adolescents with juvenile fibromyalgia. The results have shown mixed results in reducing pain and higher rates of adverse events (34). Overall, fibromyalgia management requires both pharmacological interventions and non-pharmacological interventions, as well as complementary therapies (Tai Chi, acupuncture, music, hydrotherapy, and massage therapy) and lifestyle modifications.

Neuropathic pain is a complex and debilitating condition that often requires the exploration of innovative treatment approaches to optimize patient outcomes (23, 37). Kim et al. (2011) investigated the effectiveness of pregabalin in treating neuropathic pain among 110 central post-stroke pain (CPSP) patients over 13 weeks (37). The study's results show no significant improvement in pain; however, there was improvement in sleep, anxiety, and overall patient functioning. Satoh et al. (2011) conducted a 14-week trial involving 317 Japanese patients, in which they examined pregabalin's efficacy in alleviating pain secondary to diabetic peripheral neuropathy (39). The study found a significant reduction in pain compared to the placebo group (*p* < 0.005), with some mild to moderate side effects reported. In another study, Cardenas et al. (2013) evaluated the effectiveness of pregabalin among patients with neuropathic pain associated with spinal cord injuries (38). The study found that pregabalin had a beneficial effect on reducing pain, with mild side effects. Contrary to that, in the treatment of neuropathic pain associated with HIV, Simpson et al. (2014) found no significant difference between pregabalin and a placebo (36). Yasuda et al. (2011) assessed the efficacy and safety of duloxetine in diabetic neuropathy and found it to be more effective than a placebo but noted higher adverse effects (43). Gao et al. (2012) (44) conducted a similar study on 405 Chinese patients with diabetic peripheral neuropathic pain (DPNP), which reported a significant reduction in pain and a good safety profile for duloxetine. Regarding long-term efficacy and safety, Yasuda et al. (2016) examined duloxetine in Japanese patients with DPNP and found significant pain reduction with no significant adverse effects (45). Rauck et al. (2013) investigated the optimal dose of gabapentin for treating DPNP but found no statistically significant difference between various doses and a placebo (42). We also included trials comparing multiple medications. Boyle et al. (2012) (40) evaluated the effects of amitriptyline, duloxetine, and pregabalin on DPNP, finding that all three drugs reduced pain but caused no improvement in quality of life. Padmini et al. (2012) (41) evaluated the effects of the three drugs in a study of 152 patients and reported significant pain reduction in diabetic neuropathy, with pregabalin being the fastest acting. The research studies on pregabalin, gabapentin, and duloxetine have shown positive outcomes in managing neuropathic pain, varying efficacy and tolerability across various patient populations (37–39, 42, 43, 45).

Over recent years, various clinical trials have assessed the efficacy of different medications in treating chronic low back pain (CLBP), which has led to several promising discoveries. Urquhart et al. (2016) and Donna et al. (2018) conducted randomized control trials on the use of low-dose amitriptyline in CLBP patients, showing that it may help reduce disability and pain intensity (52, 58). In 2016, Konno et al. (51) and Schukro et al. (50) studied the effectiveness of duloxetine and found significant pain reduction and improvement in secondary outcome measures. Skljarevski et al. (2009) and Williamson et al. (2014) further explored the impacts of duloxetine on pain reduction and found that patients with only a 10% reduction in pain after four weeks had a slim chance of achieving moderate pain relief after 12 weeks (48, 49). Atkinson et al. (2016) found that gabapentin had no statistically significant impact on pain relief for CLBP patients (47). Schliessbach et al. (2018) found imipramine had no overall effect on CLBP, but patients more sensitive to cold or heat pain felt better with its use (54). Atkinson et al. (2007) reported significant pain reduction and improved daily functioning in chronic back pain patients with low- and high-concentration desipramine and all-concentration fluoxetine (53). In a study conducted by Horne AW et al. (2021), gabapentin was measured in women with chronic pelvic pain and no apparent pelvic pathology (57). The study found that gabapentin did not significantly improve pain scores compared to a placebo. Lastly, Kalita et al. (2014) compared the efficacy of amitriptyline and pregabalin, finding both reduced pain and disability, but amitriptyline performed significantly better (46).

## 4 Results

### 4.1 Primary analysis

The overall summary measure (SMD (95% CI) −1.45 (−2.15, −0.75)) across all twenty studies for the difference in pain scores was statistically significant (*z* = 4.05, *p*-value < 0.001) in the intervention group when compared with the placebo. Still, there was a staggering amount of heterogeneity among the included trials (*I*^2^ = 99%). Due to their small-sized trials (*n* < 30 in each study arm), Atkinson et al. (2007) (53) and Schukro et al. (2016) (50) exhibited broader confidence intervals of the effect size. However, the intervals were narrow in large trials (Figure 1B).

### 4.2 Sensitivity analyses

**Figure 2:**
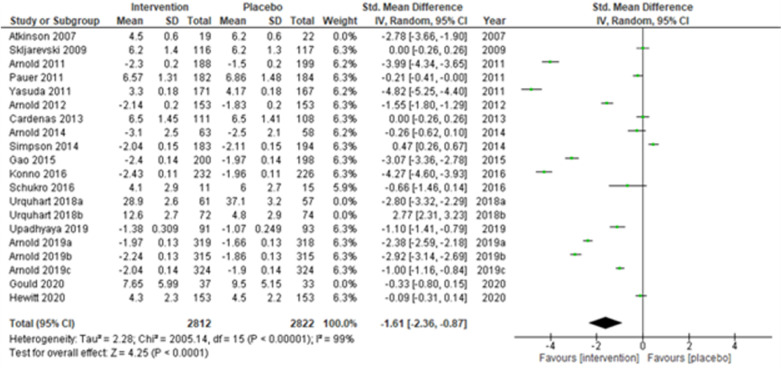
Sensitivity analysis 1—excluding studies with active placebo (benztropine).

**Figure 3:**
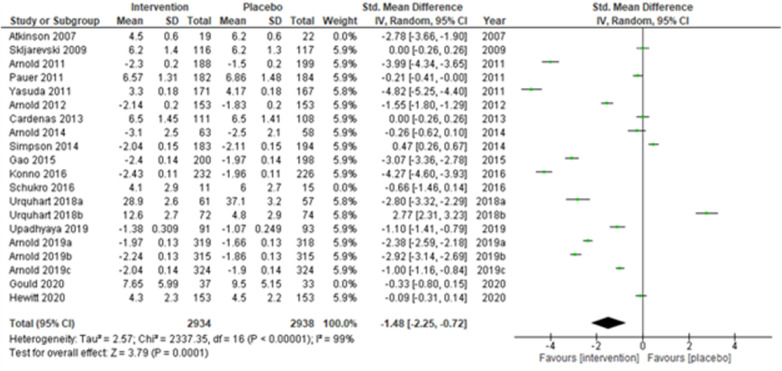
Sensitivity analysis 2—excluding studies with sample size < 50 in each study arm.

**Figure 4:**
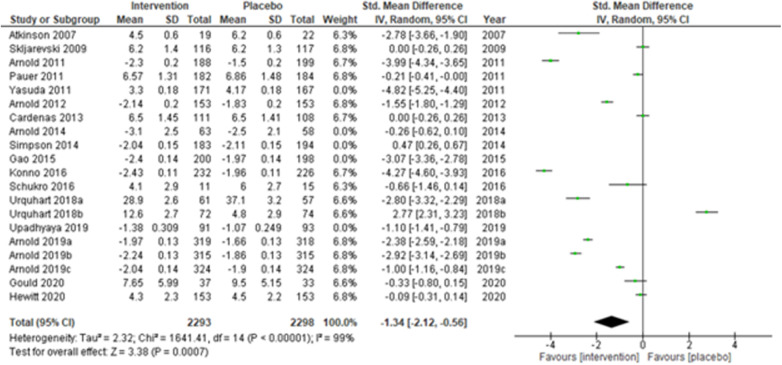
Sensitivity analysis 3—excluding studies with a high risk of bias.

We performed two to three sensitivity analyses by excluding (a) studies with active placebo, such as benztropine (52, 53) (Atkinson et al., 2007; Urquhart et al., 2018a; and Urquhart et al., 2018b); (b) studies with sample sizes < 50 in each study arm (50, 53, 55) (Atkinson et al., 2007; and Schukro et al., 2016; Gould 2020), and (c) studies with a high risk of bias (30, 34, 36, 43, 44) (Yasuda et al., 2011; Arnold et al., 2014; Simpson et al., 2014; Gao et al., 2015; and Upadhyaya et al., 2019). On excluding the effects of active placebo (Figure 2), the intervention effects (SMD (95% CI) −1.61 (−2.36, −0.87)) remained significant (*z* = 4.25, *p*-value < 0.001). Likewise, on excluding the effects of small sample-sized trials (Figure 3), the intervention effects (SMD (95% CI) −1.48 (−2.25, −0.72)) remained statistically significant (z = 3.79, *p*-value < 0.001) as well. Furthermore, the intervention effects (SMD (95% CI) −1.34 (−2.12, −0.56)) also remained statistically significant (*z* = 3.38, *p*-value < 0.001) on excluding the effects of high-risk biased studies (Figure 4). The impact of heterogeneity (*I*^2^ = 99%) did not change in any of the sensitivity analyses.

### 4.3 Sub-group analyses

**Figure 5:**
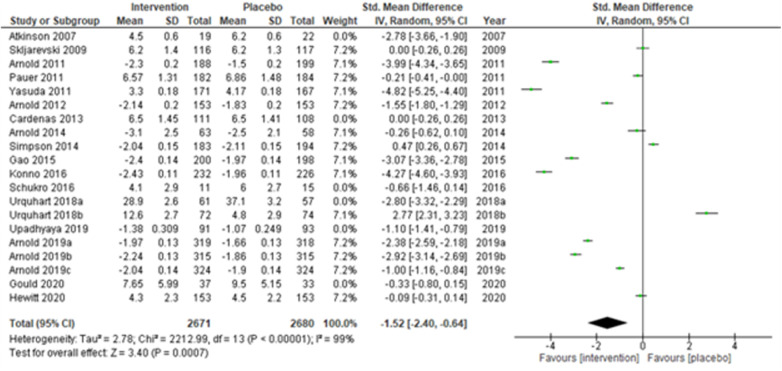
Sub-group analysis for the multicenter studies.

**Figure 6:**
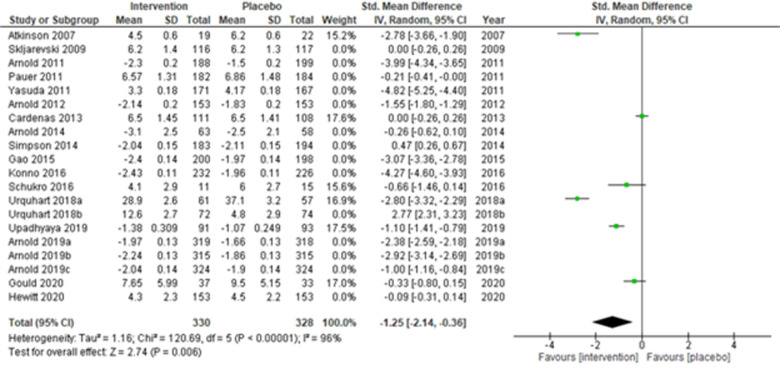
Sub-group analysis for the single-center studies.

Fourteen multi-centered studies (28, 30, 31, 33, 36, 43, 44, 48, 51, 52, 56) (Skljarevski et al., 2009; Arnold et al., 2011; Pauer et al., 2011; Yasuda et al., 2011; Arnold et al., 2012; Arnold et al., 2014; Simpson et al., 2014; Gao et al., 2015; Konno et al., 2016; Urquhart et al., 2018b; Arnold et al., 2019a; Arnold et al., 2019b; Arnold et al., 2019c; and Hewitt et al., 2020) showed significant intervention effects (SMD (95% CI) −1.52 (−2.40, −0.64); *z* = 3.40; *p*-value < 0.001; *I*^2^ = 99%) when compared with the placebo group (Figure 5). Similarly, the remaining six single-centered studies (34, 38, 50, 52, 53, 56) (Atkinson et al., 2007; Cardenas et al., 2013; Schukro et al., 2016; Urquhart et al., 2018a; Upadhyaya et al., 2019; and Hewitt et al., 2020) also showed significant intervention effects (SMD (95% CI) −1.25 (−2.14, −0.36); *z* = 2.74; *p*-value = 0.006; *I*^2^ = 96%) (Figure 6).

**Figure 7:**
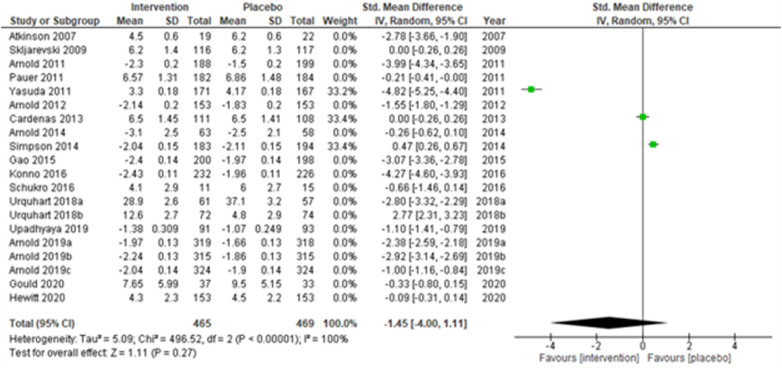
Sub-group analysis according to the type of chronic pain—neuropathic pain.

**Figure 8:**
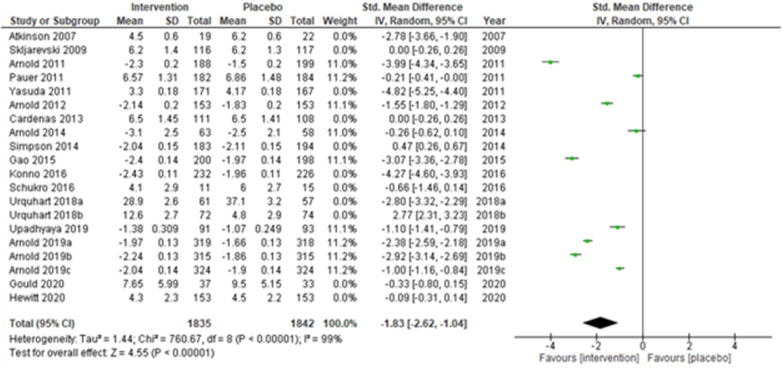
Sub-group analysis according to the type of chronic pain—fibromyalgia.

**Figure 9:**
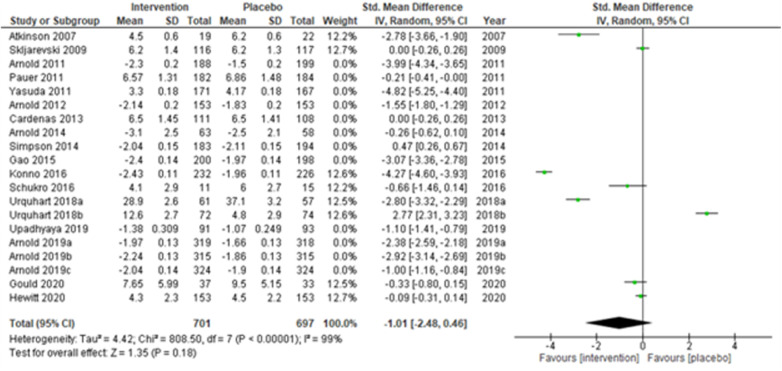
Sub-group analysis according to the type of chronic pain—chronic low back pain.

Studies were also grouped according to the type of chronic pain, such as neuropathic pain (Figure 7), fibromyalgia (Figure 8), and chronic low back pain (Figure 9). Fibromyalgia studies (28, 30–33, 44) (Arnold et al., 2011; Pauer et al., 2011; Arnold et al., 2012; Arnold et al., 2014; Gao et al., 2015; Arnold et al., 2019a; Arnold et al., 2019b; and Arnold et al., 2019c) showed significant intervention effects (SMD (95% CI) −1.83 (−2.62, −1.04); *z* = 4.55; *p*-value < 0.001; *I^2^* = 99%) when compared with placebo. Interestingly, intervention effects were found to be statistically insignificant in studies with neuropathic pain and chronic low back pain.

**Figure 10:**
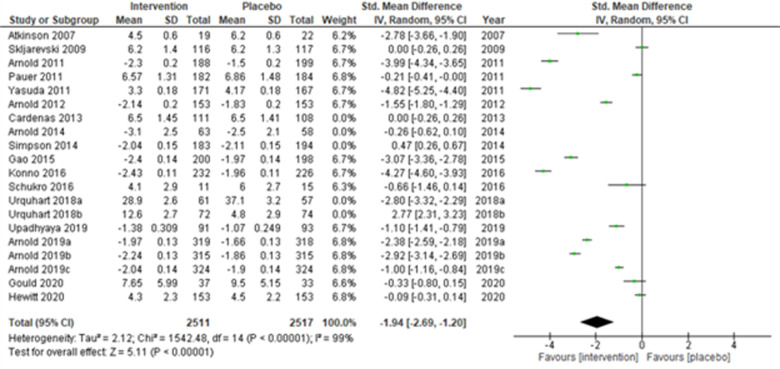
Sub-group analysis according to the study duration—short-term (≤14 weeks.).

**Figure 11:**
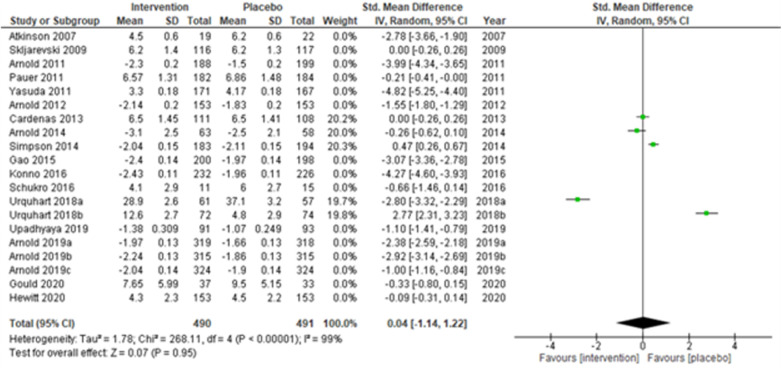
Sub-group analysis according to the study duration—long-term (>14 weeks).

Lastly, when the studies were grouped according to short-term (≤14 weeks) and long-term (>14 weeks) study duration, the present meta-analyses showed that the intervention effects in the short-term studies (28, 31–34, 43, 44, 48, 50, 51, 53, 55, 56) (Atkinson et al., 2007; Skljarevski et al., 2009; Arnold et al., 2011; Pauer et al., 2011; Yasuda et al., 2011; Arnold et al., 2012; Gao et al., 2015; Konno et al., 2016; Schukro et al., 2016; Upadhyaya et al., 2019; Arnold et al., 2019a; Arnold et al., 2019b; Arnold et al., 2019c; Gould et al., 2020; and Hewitt et al., 2020) were more statistically significant (SMD (95% CI) −1.94 (−2.69, −1.20); *z* = 5.11; *p*-value < 0.001; *I^2^* = 99%) (Figure 10) than the long-term studies (Figure 11).

### 4.4 Publication bias

**Figure 12:**
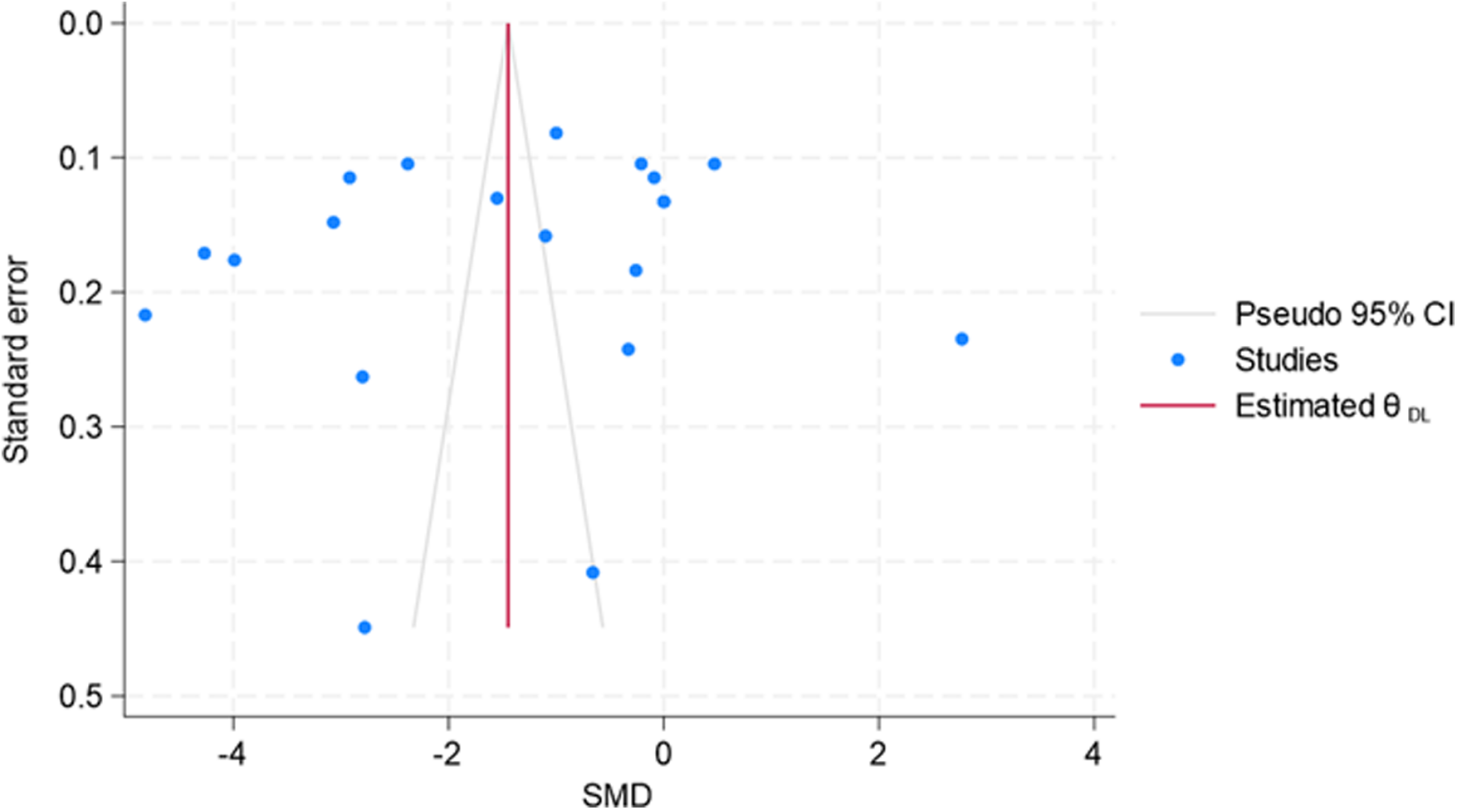
Funnel plot.

Since small-study effects are associated with publication bias, a funnel plot was used to explore the presence of small-study effects (Figure 12). Here, the study-specific effect sizes (SMD) are plotted on the *x*-axis and their standard errors (S.E.s) on the *y*-axis. The estimated $\theta_{\mathrm{DL}}$ was the summary (or pooled) treatment effect. The diagonal lines are referred to as the “pseudo 95% confidence interval” of $\theta_{\mathrm{DL}}$, which was calculated for each S.E.s and indicated the expected distribution of studies without selection bias. However, Egger's test results suggest no small-study effects (*p* = 0.442). However, due to considerable heterogeneity across the studies or outliers, the decision related to publication bias cannot be substantiated.

### 4.5 Risk of bias assessments

**Figure 13:**
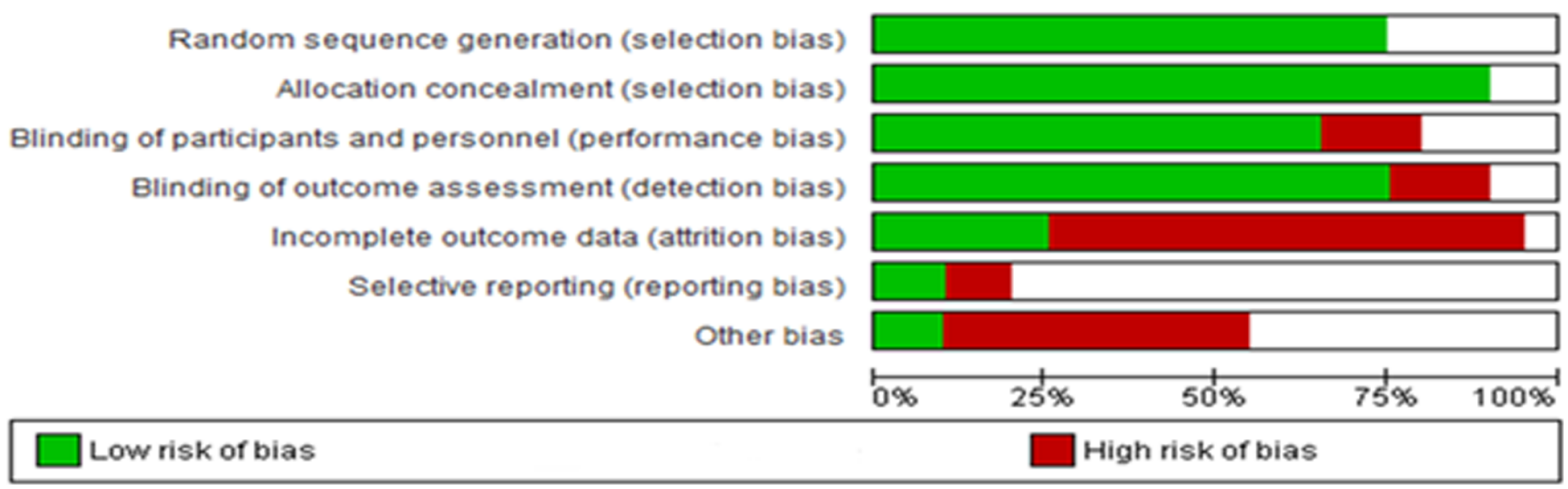
Risk of bias across all included studies. The white area suggests an unclear risk of bias.

The findings derived from the Cochrane Risk of Bias assessment tool were reported as low risk, high risk, and unclear risk (Figure 13). The majority of the studies adopted random sequence generation techniques and allocation concealment. However, most studies failed to use appropriate statistical models to accommodate missingness in the study outcomes. The risk of bias assessment indicated that most studies carried a low risk of selection, performance, and detection bias, as well as a high risk of attrition and reporting bias. Other biases were unknown.

We adopted an intention-to-treat analysis, wherein the total sample size was randomized for the intervention, and the placebo groups at the beginning of the study were considered for the present meta-analysis instead of completers. Thus, our findings minimized the effect of attrition bias.

## 5 Discussion

In our systematic review and meta-analysis, which included 20 eligible studies, we categorized these studies into three groups based on the type of chronic pain they addressed: neuropathic pain (3 studies), fibromyalgia (9 studies), and chronic low back pain (8 studies). We conducted sub-group analyses for these specific categories. Our review of 29 RCTs and meta-analysis of 20 RCTs demonstrated a significantly greater intervention effect than placebo in the fibromyalgia subgroup. However, in studies focusing on neuropathic pain and chronic low back pain, the intervention effects were statistically insignificant. This suggests that while certain psychiatric medications, such as duloxetine and mirogabalin, reduced pain in the short term (≤14 weeks), particularly for fibromyalgia, their specific effectiveness was not established for neuropathic pain and chronic low back pain. The distinction between short-term and long-term study outcomes is important for understanding the sustained impact of psychiatric medications on chronic pain. Our findings indicate that while these medications are effective in the short term, there is a need for further research into their long-term benefits and risks. This could involve studying how factors such as dosage adjustments, patient adherence, and the development of tolerance affect long-term outcomes. By addressing these factors, future guidelines could be developed to optimize the use of psychiatric medications for chronic pain management over extended periods. The long-term (>14 weeks) effects were generally insignificant. Our observation of limited long-term benefits suggests that differences in study designs, patient populations, and types of chronic pain conditions may have influenced these results.

While psychiatric medications like antidepressants and anticonvulsants are often viewed as safer alternatives to opioids, these medications have some risks as well, especially when used over the long term. Patients might experience side effects such as weight gain, sexual dysfunction, or cognitive issues. One of the long-term side effects is also the development of metabolic problems. Over time, some individuals may need higher doses to get the same pain relief, which can increase the chances of higher side effects. Additionally, stopping these medications after long-term use can sometimes cause withdrawal symptoms, which can make managing chronic pain even more challenging. Given these potential risks, it's essential for doctors to carefully consider the benefits and downsides when prescribing these medications for chronic pain. Regular check-ins, adjusting dosages as needed, and thinking about other treatment options can help minimize these side effects and ensure that patients maintain a good quality of life.

A critical factor in clinical practice is how well patients remain compliant with their prescribed psychiatric medications. Medication compliance plays a critical role in the effectiveness of these treatments, especially when managing chronic pain, where it is vital to take the medication regularly over an extended period to keep the pain controlled. In the studies we reviewed, adherence rates weren't always clearly reported, but differences in how well patients followed their treatment plans could have influenced the results. If patients are not compliant with their medication schedule, the actual effectiveness of psychiatric drugs might be underestimated since they are not getting the full benefit. On the flip side, when adherence is high, patients might see better results, but this could also lead to more side effects, which might cause some to drop out of long-term studies. Research should look at ways to boost adherence, like better patient education, simpler dosing schedules, and regular check-ins, to ensure patients get the most out of these treatments.

Several published studies investigated the use of non-opioid psychiatric medications for managing chronic pain. McDonagh et al. (2020) performed a comparative review to explore the effectiveness of non-opioid treatments for chronic pain across various durations (short-term 3 to <6 months, intermediate-term 6 to <12 months, and long-term ≥ 12 months) (20). McDonagh et al. (2020) reported minor improvements in pain and function with duloxetine for chronic low back pain, where our study did not find a statistically significant effect for this condition. The high heterogeneity in our study, particularly in the chronic low back pain subgroup, may explain this discrepancy. Both studies, however, emphasize the limited evidence for long-term efficacy of these medications. As McDonagh et al. highlighted the need for careful interpretation of non-opioid treatments for chronic pain (20), our findings similarly underscore the importance of further research, particularly in identifying specific subgroups that might benefit more from psychiatric medications.

Ferreira et al. (2021) conducted a systematic review and meta-analysis examining the efficacy and safety of antidepressants in treating back pain and osteoarthritis pain (24). The authors found that selective serotonin-norepinephrine reuptake inhibitors (SNRIs) such as duloxetine and milnacipran may offer short-term pain reduction for these conditions within 3–13 weeks. However, they noted that the effect on back pain was clinically insignificant, though a clinically important effect could not be excluded for osteoarthritis. Regarding sciatica pain, they observed a very low certainty of evidence for SNRIs reducing pain within the first two weeks, with no sustained effect beyond this period. For tricyclic antidepressants (TCAs), their study found that while TCAs were not effective for back pain, they showed some efficacy in reducing sciatica pain in the short and medium term (3–12 months). In contrast, our research specifically examined the efficacy of TCAs (Amitriptyline and Desipramine) for chronic low back pain and found no statistically significant effects. For Amitriptyline, the effect size was almost zero with an extensive confidence interval. Similarly, Desipramine's effect size also suggested no clear benefit for chronic low back pain, with a wide confidence interval that included the possibility of no effect. Although Ferreira et al. focused on back pain and osteoarthritis, Caruso et al. (2019) evaluated the role of antidepressants in treating psychiatric symptoms like depression and anxiety in patients with neuropathic pain (25). These conditions are often comorbid with chronic pain. The authors found that antidepressants were more effective than placebo in reducing depressive symptoms (SMD = −0.11; NNT = 24), though their effect on anxiety was insignificant. Their study shows the improvement in quality of life, which suggests that managing psychiatric symptoms may play an important role in the overall treatment of neuropathic pain. This highlights the broader utility of antidepressants in chronic pain management, especially for patients who experience comorbid psychiatric conditions.

Regarding TCAs, Urquhart et al. 2008 (62) found no clear evidence of their effectiveness in chronic low-back pain management. However, In a later study, Urquhart et al. 2018 suggested that low-dose amitriptyline could reduce disability at three months, although there were no significant improvements in pain reduction or disability at six months (52). Schliessbach et al. (2018) evaluated the effect of the tricyclic antidepressant imipramine on chronic low-back pain and found no significant immediate analgesic effect (54). However, they noted that anti-nociceptive effects might depend on the CYP2D6 genotype, suggesting that metabolizer status should be considered in future studies with tricyclic antidepressants. These findings resonate with our observations, underscoring the complexity of treatment responses and the potential influence of genetic factors on the efficacy of TCAs in chronic low back pain. The systematic review and meta-analysis conducted by Finnerup et al. in 2015 investigated the efficacy of various pharmacotherapies for treating neuropathic pain, including medications from the gabapentinoids family (23). This study found moderate effectiveness for drugs like SNRIs such as duloxetine and venlafaxine (NNT 6.4, 95% CI of 5.2–8.4), pregabalin (NNT 7.7, 95% CI of 6.5–9.4), and gabapentin (NNT 6.3, 95% CI of 5.0–8.3). The study also noted that tricyclic antidepressants had lower NNTs compared to SNRIs and gabapentinoids, making them a potentially more effective first-line treatment for neuropathic pain (23). Additionally, some recent trials conducted in Japan and the U.S. with 40 and 60 mg of once-daily duloxetine further strengthen duloxetine as an effective agent for diabetic neuropathic pain (43–45).

Our study aimed to assess the therapeutic efficacy of gabapentinoids for treating fibromyalgia and various types of neuropathic pain. Our results provide new insights into the role of these drugs in managing chronic pain conditions. Our findings on pregabalin showed a statistically significant improvement in pain scores for fibromyalgia, which aligns with previous research on its use in chronic pain conditions. However, as noted in our meta-analysis, the effect size was modest, suggesting that while statistically significant. The clinical impact such finding may be limited, particularly when weighing potential side effects and patient preferences. Our study investigated the impact of Mirogabalin, and it emerges as a viable treatment option for fibromyalgia. The important effect size, represented by an SMD of −2.10, which suggests its clinical significance, potentially leading to a noticeable improvement in patients’ symptoms. Our research observed a small to moderate average effect size for pregabalin in treating neuropathic pain. However, the overall effect was not statistically significant. As for duloxetine, only one study was available for analysis, making a meta-analysis inapplicable. Thus, pregabalin may have limited clinical significance in treating neuropathic pain, while the effectiveness of duloxetine remains unclear.

The findings of this systematic review highlight a noticeable short-term benefit of certain psychiatric medications, particularly Duloxetine and Mirogabalin for the treatment of fibromyalgia. However, the lack of consistent evidence supporting the long-term efficacy of these drugs requires a cautious approach to their use in chronic pain management. The marked heterogeneity across studies warrants careful consideration of individual patient factors, including psychiatric comorbidities, while assessing the appropriateness of these treatments. It also highlights the need for future research to better understand the complexities of chronic pain and its treatment, with the goal of improving patient selection and optimizing treatment durations for better outcomes. While the current analysis does not endorse the widespread application of these medications across all forms of chronic pain, the substantial short-term efficacy observed in fibromyalgia patients offers a viable non-opioid alternative that may be particularly beneficial in contexts where opioid use is contraindicated or poses a significant risk.

## 6 Limitation

Our study has several limitations that should be considered. our systematic review only included randomized controlled trials (RCTs). While this decision enhances the quality of the included studies, it may lead to the exclusion of quality clinical trials that do not employ an RCT design, potentially introducing bias to our findings. This meta-analysis was conducted across different disease categories, such as fibromyalgia, neuropathic pain, and chronic back pain. This approach allowed us to assess the overall effectiveness of non-opioid psychiatric pharmacotherapies across different pain domains, which was the primary objective of our study. However, our analysis does not offer a detailed comparison of the effectiveness of specific medication types or dosages. Our study focused solely on psychoactive non-opioid pharmacotherapies, such as SNRIs and TCAs, and excluded non-psychoactive treatments like NSAIDs, lidocaine patches, or capsaicin. While these non-psychoactive options could provide valuable insights into pain management, they were not within the scope of our analysis.

## 7 Conclusion

This study evaluates the efficacy of various psychiatric medications in pain management. We found that duloxetine and mirogabalin were significantly effective in the short-term treatment of fibromyalgia. However, the effectiveness of these medications, as well as others such as amitriptyline, desipramine, and gabapentin, remains uncertain for mixed neuropathic conditions and chronic low back pain remains uncertain due to inconsistent results and limited data, as indicated by published literature. While some evidence supports the use of these medications in specific types of neuropathic pain, such as diabetic neuropathy or postherpetic neuralgia, our analysis did not find consistent long-term benefits for the treatment of chronic pain conditions. The lack of consistent long-term benefits for these treatments highlights the need for further research into both the short- and long-term effects of psychiatric medications in chronic pain management. Clinicians should interpret these findings with caution, considering the individual characteristics and clinical contexts of their patients. A comprehensive, interdisciplinary approach that integrates both pharmacological and non-pharmacological strategies remains vital in managing chronic pain effectively.

## Data Availability

The original contributions presented in the study are included in the article/Supplementary Material, further inquiries can be directed to the corresponding author.
